# Targeting CRTC2 reverses *STK11* mutant NSCLC tumor resistance to immunotherapy

**DOI:** 10.1073/pnas.2508762123

**Published:** 2026-04-22

**Authors:** Dimitri Robay, Ole Ackermann, Laurent Laborde, Xingyi Shi, Federico Oreglia, Ramona Stump, Sabina Ciaghi, Giorgio G. Galli, Laura Holzer, Lorraine Villemin, Joel Wagner, Jincheng Wu, Lang Ho Lee, Claudia Bossen, Thanos P. Mourikis, Millicent Gabriel, David Ruddy, Haiyan Yu, Cory Johannessen, Stephane Ferretti, Carlotta Costa

**Affiliations:** ^a^Novartis Biomedical Research, Oncology, Basel 4056, Switzerland; ^b^Novartis Biomedical Research, Oncology, Cambridge, MA 02139; ^c^Novartis Pharma, Basel 4056, Switzerland

**Keywords:** lung cancer, STK11, LKB1, ICB resistance, CRTC2

## Abstract

Non–small cell lung cancer (NSCLC) patients with tumors harboring *STK11* mutations are resistant to standard of care anti-PD-1/PD-L1 blockade. For this patient population there are no currently available tailored treatments, underlying the critical need to discover effective therapeutic strategies. In this study, we dissected the molecular mechanisms responsible for *STK11*-mediated resistance to immune checkpoint blockade (ICB) and identified CRTC2, a coactivator of the transcription factor cAMP response element-binding protein (CREB), as a key signaling node regulating *Stk11*-dependent cell-extrinsic functions. CRTC2 deletion remodeled the immune profiles of *Stk11*-KO tumors and resensitized them to anti-PD-1 treatment, comparably to *Stk11*-proficient tumors. Mechanistically, the abrogation of the binding between CRTC2 and CREB was sufficient to restore sensitivity to immunotherapy. These findings provide critical insights into the central role of CRTC2 in modulating response to ICB and identify the disruption of CRTC2–CREB interaction as a potential therapeutic approach for this patient population.

## Significance

Inactivating mutations of the *STK11* gene, found in about 15 to 20% of non–small cell lung cancer (NSCLC) patients, are predictive of clinical resistance to PD-1/PD-L1 inhibitors and are associated with changes in the tumor immune microenvironment. In this manuscript, we uncovered the mechanism exploited by *STK11* mutant NSCLC tumors to dampen the response to immunotherapy and identify CRTC2 as a key regulator in this process. Therefore, targeting CRTC2 may offer a promising therapeutic strategy for this patient population refractory to current standard of care treatments.

Despite anti-PD-1/PD-L1 therapies becoming a component of standard of care treatment for several oncology indications, including non–small cell lung cancer (1), a large fraction of patients does not respond to these treatments, highlighting the necessity to discover biomarkers associated with primary resistance, underlying resistance mechanisms, and strategies to overcome them.

Multiple studies have shown that mutations in the *STK11* gene are predictive of clinical resistance to PD-1/PD-L1 blockade (2–4) and are associated with low PD-L1 expression and low T cell infiltration into the tumors (2, 5, 6). *STK11* inactivating mutations occur in approximately 15 to 20% of patients with NSCLC (7), making *STK11* the third most frequent genetic alteration in this patient population after *TP53* and *KRAS*. *STK11* encodes for LKB1, a serine/threonine kinase that regulates 13 downstream kinases (8) and plays a pleiotropic role by regulating a variety of cellular processes, such as cell polarity, cell growth, and metabolism (9). It has been elucidated that among the 13 substrates, SIK subfamily members are necessary in mediating the tumor-intrinsic suppressive effects of LKB1 (10, 11). In addition, CRTC2, an established target of the SIKs and a transcriptional coactivator of the CREB transcription factor, impacts in vitro proliferation (10) and tumor initiation and establishment (12) of NSCLC *STK11* mutant models in immunocompromised mice. While in the last years research efforts have focused on uncovering the signaling nodes mediating LKB1-tumor intrinsic suppressive functions, the mechanisms regulating its tumor immune-evasive functions still remain unknown, and a targeted therapeutic approach for NSCLC patients with *STK11* mutant tumors represents an unmet medical need for this patient population.

## Results

**Fig. 1.**
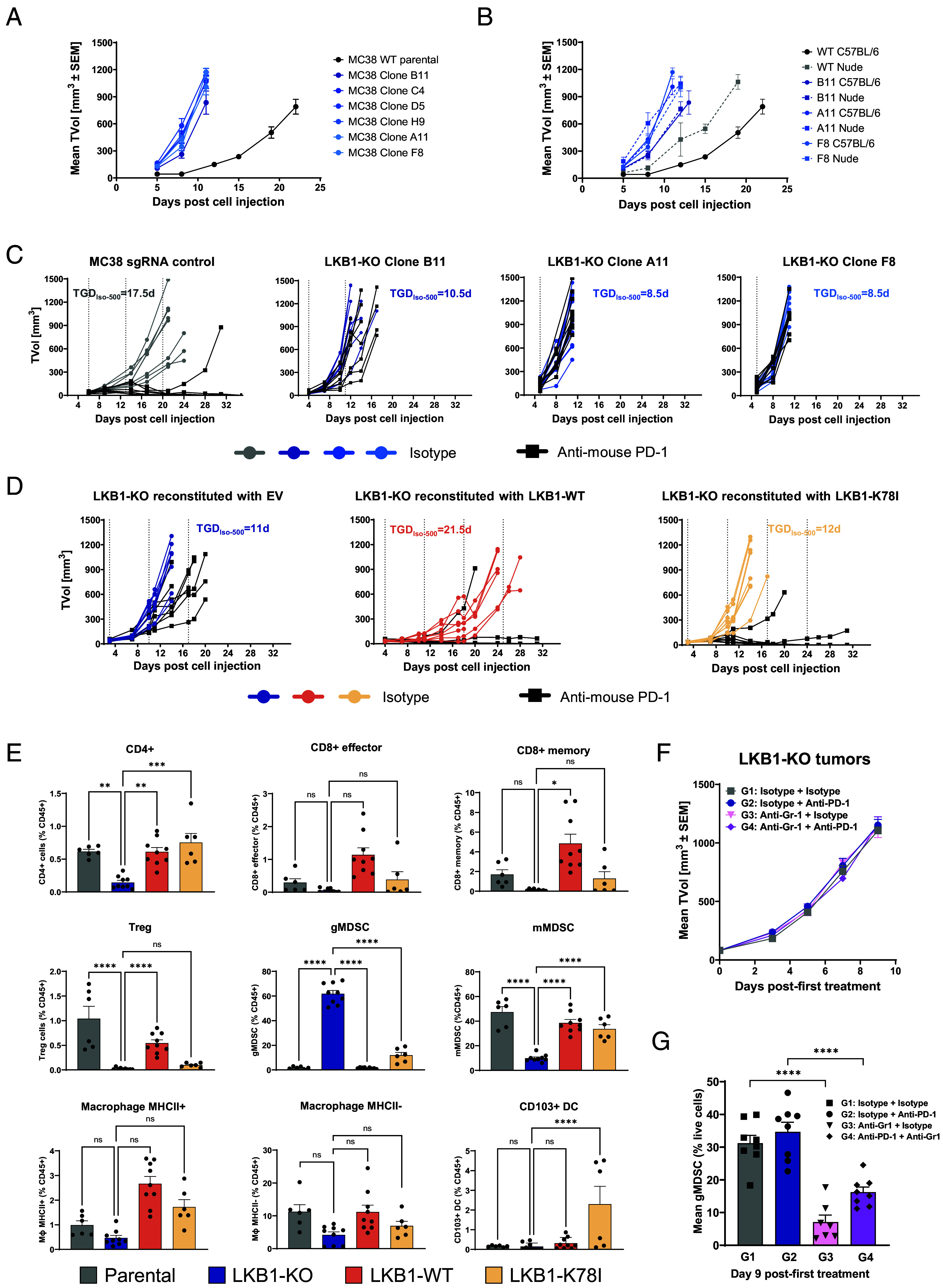
LKB1 loss causes TME remodeling and insensitivity to immunotherapy. (*A* and *B*) Tumor volumes of LKB1-KO and WT MC38 cells were measured in C57BL/6 mice (*A*) or C57BL/6 and Nude mice (*B*). Data are presented as the mean ± SEM. (*C* and *D*) Tumor volumes of MC38 cells with indicated genotypes were treated with either isotype control or anti-PD-1 antibody 1D2. Data are presented as individual tumor growth. Vertical dotted lines define the treatment time-points. TGD_Iso-500_ is the tumor growth delay to reach 500 mm^3^ for each individual tumor in the isotype treatment group. EV = empty vector. (*E*) Quantification of intratumoral immune populations from isotype-treated tumors with indicated genotypes (depicted as percentage of CD45^+^ cells). Data are presented as the mean± SEM; statistical difference was assessed by using one-way ANOVA with Sidak’s multiple group comparison test. (*F*) Tumor volumes of LKB1-KO MC38 cells treated with either isotype control, anti-Gr-1 antibody, anti-PD-1 antibody, or the combination. Data are presented as the mean ± SEM. (TVol: Tumor volume). (*G*) Quantification of intratumoral gMDSC cells from tumors in (*F*). Data are presented as the mean ± SEM; statistical difference was assessed by using one-way ANOVA with Sidak’s multiple group comparison test.

To assess the relationship between LKB1 and the lack of response to anti-PD-1 therapy, we decided to utilize the immune-competent colorectal cancer model MC38, which is sensitive to anti-PD-1 therapy (13) and therefore suitable to assess potential mechanisms of resistance. This is because commercially available immune-competent NSCLC models, such as LLC and FVBW-17 bearing wild-type *Stk11* alleles, do not respond to anti-PD-1 therapy (14, 15). Notably, colorectal syngeneic models have been effectively employed to model LKB1-related clinical findings and biology (3). We generated a panel of LKB1 knock-out (KO) clones (*SI Appendix,* Fig. S1*A*) and observed that all LKB1-KO clones grew faster than MC38 control tumors in vivo (Fig. 1*A*). We then compared their growth in athymic and C57BL/6 mice to evaluate if this was related to LKB1-mediated cell-extrinsic immune-modulation. LKB1-KO tumors grew faster than controls also in nude mice (Fig. 1*B*), indicating that LKB1 affects aggressiveness of tumor growth independently of the immune system, in accordance with its well-established cell-intrinsic role as tumor suppressor (16). While control tumors grew slower in C57BL/6 mice in comparison to nude mice, likely due to an immune-mediated reaction against the tumor, this difference between strains was lost in LKB1-KO tumors (Fig. 1*B*), suggesting that in immunocompetent mice the loss of LKB1 can induce evasion of the immune system. To then characterize the causality of LKB1 in resistance to ICB, we challenged a subset of our LKB1-KO clones with anti-PD-1 treatment. Consistent with the impact of LKB1 loss on immune escape (Fig. 1*B*), every LKB1-KO clone tested was insensitive to anti-PD-1 treatment, in contrast to LKB1-proficient MC38 control tumors (Fig. 1*C*). To investigate this finding further, LKB1-KO cells were reconstituted with either the wild-type or the K78I hypomorphic mutant alleles of LKB1 (*SI Appendix,* Fig. S1*B*). Of note, the LKB1-K78I isoform has been historically described as kinase-dead and indeed it phenocopied LKB1-deficient models in respect of LKB1 cell-autonomous functions (17). However, it has been recently demonstrated that it retains 10% of the wild-type kinase activity, qualifying it as a hypomorphic mutant (18) (*SI Appendix,* Fig. S1*B*). While the reexpression of LKB1-WT slowed down tumor growth and resensitized tumors to anti-PD-1 therapy, the expression of LKB1-K78I did not impact the growth rate of tumors but resensitized the tumors to anti-PD-1 therapy (Fig. 1*D*), suggesting that partial activity of LKB1 was sufficient to modulate its immune-mediated functions. Additionally, LKB1 had a major impact on the immune composition of the tumors. LKB1-KO tumors showed a significant gMDSC (granulocytic myeloid-derived suppressor cells) infiltration in comparison to controls, in addition to a decrease in the number of mMDSC (monocytic myeloid-derived suppressor cells), Treg and CD4^+^ T cells, and almost a complete loss of CD8^+^ T cells (both effector and memory, although the impact did not reach statistical significance) (Fig. 1*E* and *SI Appendix,* Fig. S1*C*). Interestingly, tumors expressing the hypomorphic version of LKB1 showed an intermediate immune phenotype.

Recently, LKB1 loss was associated with increased gMDSC tumoral abundance in multiple preclinical models (3, 5, 19). Moreover, in patients with *STK11* mutant tumors, an increased neutrophil density (4, 20), and an increased expression of cytokines associated with neutrophilic contexture (3) were observed. Therefore, we decided to investigate how loss of LKB1 impacts gMDSC biology. Tumor-derived MDSC showed a similar T cell suppression activity (*SI Appendix,* Fig. S1 *D* and *E*), and ability to migrate toward cancer cells (*SI Appendix,* Fig. S1*F*–blue vs. orange columns), regardless of the genotype of the tumors from where they were extracted, suggesting that LKB1 does not impact the functionality of MDSCs. However, LKB1-KO cancer cells showed increased capacity to attract MDSC (*SI Appendix,* Fig. S1*F*–plain vs. striped columns), suggesting that this is the underlying cause of the increased gMDSC recruitment into LKB1-deficient tumors. Next, we assessed if MDSC elimination was sufficient to resensitize LKB1-deficient tumors to anti-PD-1 treatment. Even if depletion of both gMDSC and mMDSC with anti-Gr-1 antibody was initially very efficient (*SI Appendix,* Fig. S1 *G* and *H*), this had no effect on the response to anti-PD-1 treatment (Fig. 1*F*). These data suggest that ablation of MDSCs is not sufficient to restore the sensitivity to anti-PD-1 therapy in this model. Importantly, MDSC depletion was not sustained over time (Fig. 1*G* and *SI Appendix,* Fig. S1*I*), in line with the previously reported “rebound” effect (21, 22) and potentially limiting the interpretation of the results.

**Fig. 2.**
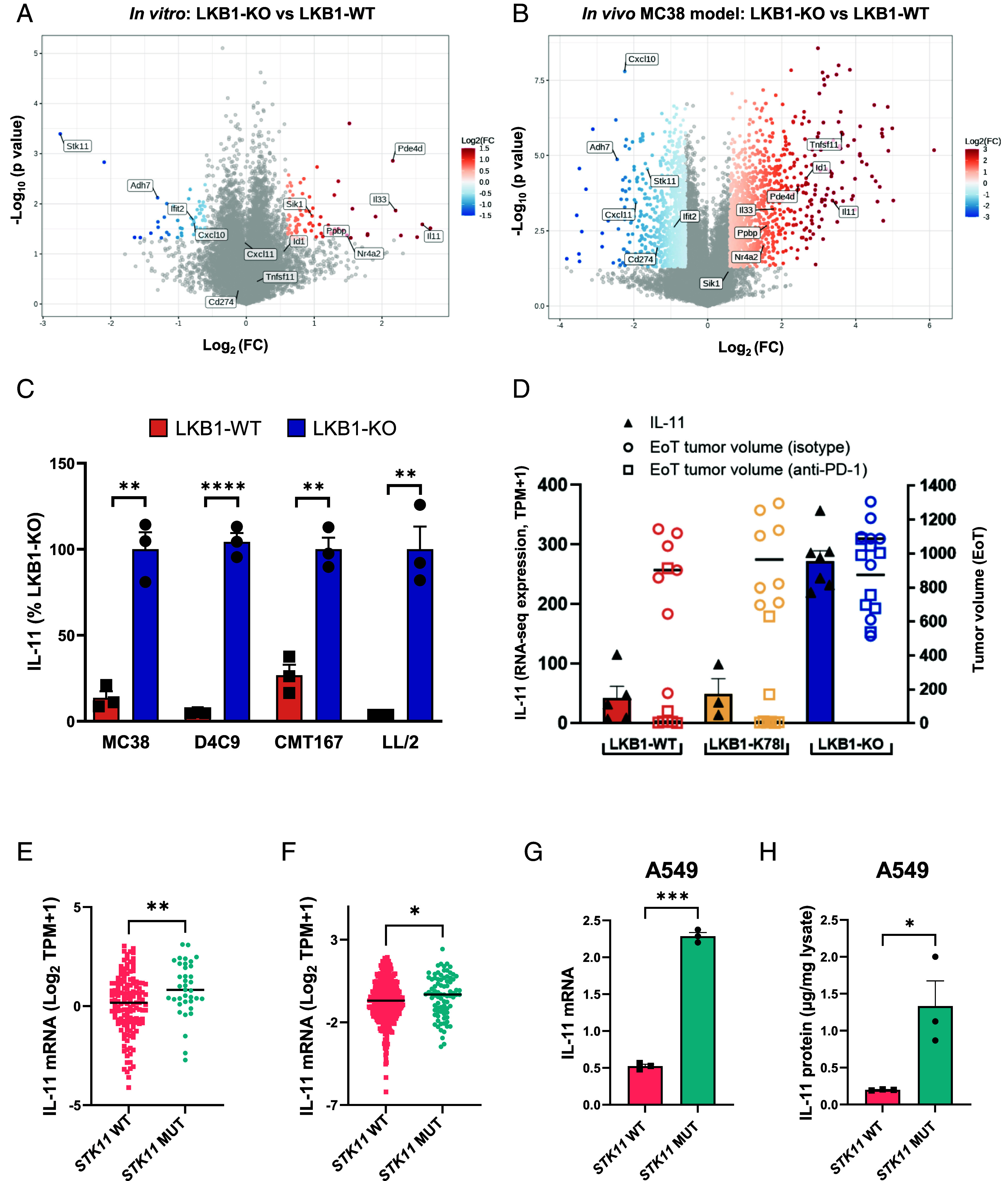
LKB1 loss induces extensive transcriptional reprogramming. (*A* and *B*) Volcano plots showing the comparisons of LKB1-KO and LKB1-WT cell lines (*A*), and cancer cells isolated from LKB1-KO and LKB1-WT MC38 tumors (*B*). *x*-axis represents the logarithmic 2 scaled fold-change (FC) between the average of indicated samples; *y*-axis depicts the negative logarithmic 10 scaled *P*-value. Genes are colored based on significant Log2(FC) (*P*-value < 0.05). (*C*) IL-11 levels were quantified by ELISA from the supernatants of indicated cell lines. For normalization, the mean of the LKB1-KO group of each cell line was set to 100%. Data are presented as the mean ± SEM. Statistical difference was assessed by the *t* test. (*D*) Scatter plot representing IL-11 mRNA levels (left *y*-axis and black triangles) and tumor volumes at the end of treatment (EoT) (right *y*-axis and colored circles/squares) for tumors in Fig. 1*D*. *X*-axis groups data according to the genotypes. (*E*) Dot plot representing IL-11 mRNA levels in *STK11* wild-type (WT) and mutant (MUT) NSCLC cell lines from CCLE (24). Statistical difference was calculated by using the Mann–Whitney *U* test. (*F*) Dot plot representing IL-11 mRNA levels from in silico deconvoluted cancer cells in *STK11* wild-type (WT) and mutant (MUT) tumors using TCGA LUAD dataset. Statistical significance was tested by Wilcoxon-test. (*G* and *H*) mRNA quantified by qRT-PCR (*G*) and protein (quantified by ELISA) (*H*) for IL-11 levels from the *STK11* mutant (MUT) and wild-type (WT) isogenic pair of A549 cells. Data are presented as the mean ± SEM. Statistical difference was calculated by the *t* test.

These data suggest that multiple immune populations beyond MDSC likely play a role in regulating the sensitivity to immunotherapy. LKB1 affects the expression of many secreted factors known to have a pleiotropic effect on the TME (tumor microenvironment) (3, 5, 19). To investigate LKB1-associated transcriptional changes, the expression profile of four LKB1-KO murine isogenic pairs models was analyzed (Fig. 2*A* and *SI Appendix,* Fig. S2 *A*–*D*). Among the top upregulated genes, we found genes such as *Pde4d, Il33, Ppbp, Il11*, and *Nr4a2*, previously reported to be modulated by LKB1, both in human and murine cell lines (10, 12, 19, 23). At the transcriptional level, cells expressing the kinase-dead mutant LKB1-D194A were more comparable to LKB1-KO cells, while the hypomorphic LKB1-K78I (which retains partial kinase activity) showed an expression pattern more similar to LKB1-WT (*SI Appendix,* Fig. S2 *E*–*G*), suggesting that the ability of LKB1-K78I to rescue ICB resistance is related to its partial retention of the kinase activity, rather than LKB1-dependent scaffolding function. The pattern of transcriptional modulation was validated in cancer cells isolated from LKB1-KO and WT MC38 tumors (Fig. 2*B*). Interestingly, the regulation of some genes such as *Tnfsf11*, *Id1*, *Cxcl11,* and *Cd274* (which encodes for PD-L1), was apparent only in vivo, suggesting that their expression is modulated by secreted factors not present in cultured conditions. The soluble factor IL-11 was among the top genes upregulated in LKB1-deficient models both in vitro and in vivo (Fig. 2 *A* and *B*) and was the focus of further validation studies. Increased expression of IL-11 was confirmed at the protein level across different models, including NSCLC *KRAS* mutant cell lines CMT167 and LL/2 (Fig. 2*C*). Moreover, IL-11 levels, either in serum (*SI Appendix,* Fig. S2*H*) or in cancer cells isolated from MC38 tumors (Fig. 2*D*) anticorrelated with response to anti-PD-1 treatment. Accordingly, IL-11 expression was higher in *STK11* mutant human NSCLC cell lines (Fig. 2*E*) and tumors (Fig. 2*F*) in comparison to wild-type counterparts. These results were validated both at RNA and protein levels in an isogenic pair derived from the human NSCLC cell line A549, naturally deficient for LKB1 expression (Fig. 2 *G* and *H*). Since *Stk11*-dependent cell-extrinsic functions are likely mediated by a plethora of genes (Fig. 2 *A* and *B*), IL-11 alone is likely not sufficient to modulate the TME and sensitivity to anti-PD-1. In the following studies therefore IL-11 was utilized only as representative biomarker for LKB1 activity, without functional implications.

**Fig. 3.**
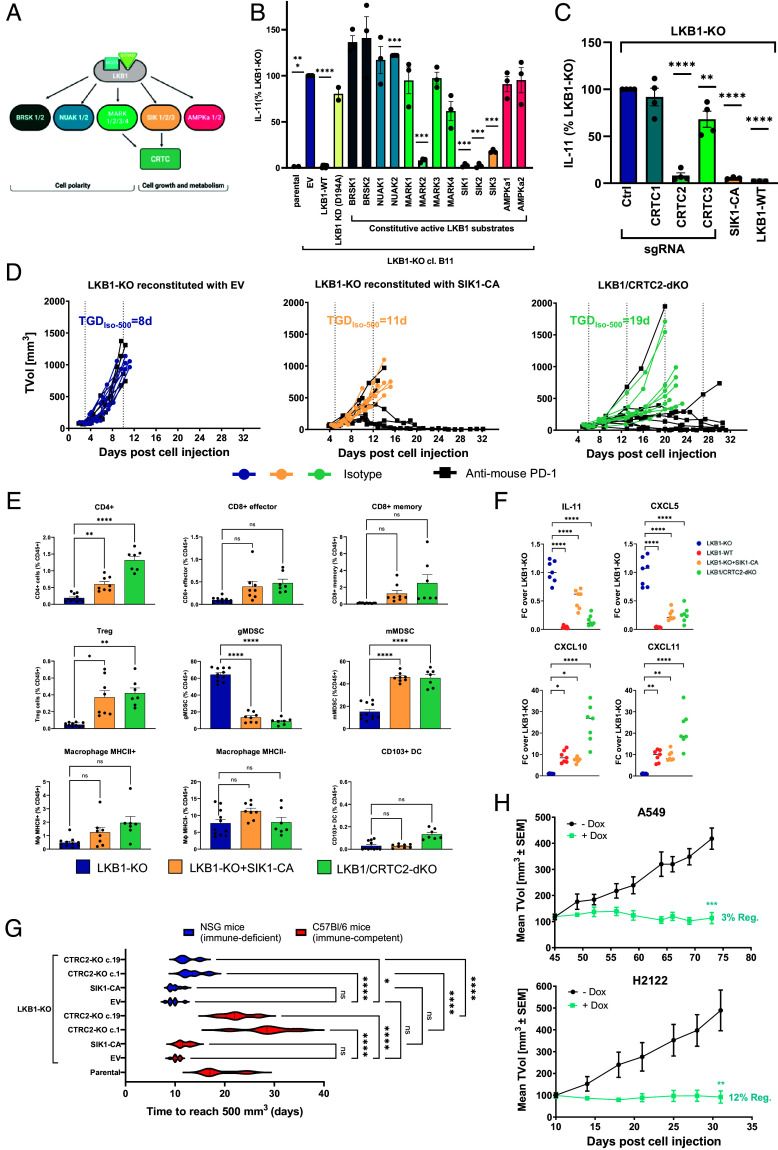
CRTC2 and SIK are key signaling nodes regulating LKB1-dependent functions. (*A*) Schematic cartoon of LKB1 family substrates. (*B* and *C*) IL-11 levels were quantified by ELISA from the supernatants of MC38 cells with indicated genotypes. EV = empty vector. For normalization, the LKB1-KO value of each experiment was set to 100%. Data are presented as the mean ± SEM. Statistical difference was assessed with a one-way ANOVA with Dunnett’s multiple group comparison post hoc test. (*D*) Tumor volumes (TVol) of MC38 cells with indicated genotypes were treated with either isotype control or anti-PD-1 antibody. Vertical dotted lines define the treatment time-points. EV = empty vector. Data are presented as individual tumor growth. TGD_Iso-500_ is the tumor growth delay to reach 500 mm^3^ for each individual tumor in the isotype treatment group. (*E*) Quantification of intratumoral immune populations from isotype-treated tumors with indicated genotypes (depicted as percentage of CD45^+^ cells). Data are presented as the mean ± SEM; statistical difference was assessed by using one-way ANOVA with Sidak’s multiple group comparison post hoc test. (*F*) Scatter plots representing qRT-PCR of selected genes on cancer cells isolated from isotype-treated tumors presented in (*D*) and LKB1-WT tumors in Fig. 1*D*. Individual points represent different tumors, and the bar represents the median value. For normalization, the mean of the LKB1-KO group was set to 1. Statistical difference was calculated by one-way ANOVA with Dunnett’s multiple group comparison test. (*G*) Violin plot representing the time for MC38 tumors with indicated genotypes to reach 500 mm^3^. EV = empty vector. Statistical difference was assessed with a one-way ANOVA with Sidak’s multiple group comparison test. (*H*) Tumor volumes from A549 (*Top*) and H2122 (*Bottom*) cells transduced with inducible shRNA against CRTC2 and measured in mice treated with 10% sucrose or doxycycline. Data are presented as the mean ± SEM. Statistical differences in tumor volumes (TVol) at the end of treatment were assessed by using an unpaired *t* test.

To identify which of the 13 kinases downstream of LKB1 (Fig. 3*A*) mediates the LKB1-dependent cell-extrinsic phenotype, we generated for each kinase a phospho-mimetic mutant by a single threonine to glutamic acid substitution in the T loop that renders the kinase constitutively active (8). Only the expression of constitutively active SIK1/2/3 and MARK2 reduced IL-11 levels to a similar extent as the LKB1-WT construct (Fig. 3*B* and *SI Appendix,* Fig. S3*A*). Among the signaling effectors further downstream, the CRTC (cAMP-regulated transcriptional coactivator) family members are regulated both by SIK and MARK (Fig. 3*A*). CRTC1/2/3 were found in an unbiased screen as the most potent coactivators of the transcription factor CREB (25). Among the three family members, only the knock-out of CRTC2, which is the most highly expressed isoform in NSCLC (12), suppressed IL-11 production comparably to LKB1 reexpression (Fig. 3*C* and *SI Appendix,* Fig. S3 *B* and *C*). These findings were validated in NSCLC *KRAS* mutant cell lines CMT167 and LL/2 (*SI Appendix,* Fig. S3 *D* and *E*). SIK and MARK can phosphorylate CRTC2, and this event induces CRTC2 binding to 14-3-3 and its retention in the cytoplasm (26, 27). In the absence of LKB1, this phosphorylation does not occur and CRTC2 translocates into the nucleus where it binds CREB and regulates transcriptional activity (*SI Appendix,* Fig. S3*F*). After reexpression of LKB1 in LKB1-KO cancer cells, we observed an upper shift in the migration of CRTC2 band (*SI Appendix,* Fig. S3*G*), likely related to its phosphorylation in the presence of LKB1. Both the expression of the constitutively active form of SIK1 (SIK1-CA) and the knock-out of CRTC2 (CRTC2-KO) resensitized LKB1-deficient tumors to anti-PD-1 therapy (Fig. 3*D*). In addition, CRTC2-KO and SIK1-CA tumors showed an increase of CD4^+^, Treg, and mMDSC populations, and a reduction of gMDSC in comparison to LKB1-KO tumors (Fig. 3*E*). Both SIK2-CA and SIK3-CA tumors exhibited an immunophenotype closely resembling that observed with SIK1-CA (*SI Appendix,* Fig. S4*A*). These immune phenotypes resemble those observed in LKB1-proficient tumors (Fig. 1*E*). Consistently, ablation of CRTC2 and expression of SIK1-CA in a LKB1-null context induced the expression of several secreted factors similarly to LKB1-WT tumors (Fig. 3*F*). All together, these findings indicate that SIK and CRTC2 are key signaling nodes that regulate LKB1-dependent sensitivity to immunotherapy.

CRTC2/LKB1 double KO (dKO) tumors grew significantly slower than LKB1-KO tumors in the isotype-treated arm (Fig. 3*D*). To assess if this was linked to an immune-mediated or rather a cell-intrinsic function, tumors were grown in both immune-competent and immune-deficient mouse strains (Fig. 3*G*). While the expression of the constitutively active form of SIK1 did not have an impact on tumor growth, CRTC2-deficient tumors grew significantly slower in immune-deficient mice (Fig. 3*G*–blue violin plots), suggesting that CRTC2 has also a cell-intrinsic mechanism in addition to the immune-modulated mechanism observed in immune-competent mice (Fig. 3*G*–red violin plots), as previously reported for the LKB1–CRTC2 axis in a context of tumor initiation and establishment (12, 28). The impact of CRTC2 however is significant only in a LKB1-KO context since CRTC2-KO tumors in a LKB1-proficient MC38 background exhibit similar growth rate than control tumors (*SI Appendix,* Fig. S4 *B* and *C*). To assess if CRTC2 depletion also impacts the growth of already established tumors (tumor maintenance), we engineered two NSCLC *STK11*mutant models with an inducible shRNA against human CRTC2 (*SI Appendix,* Fig. S4*D*). Knock-down of CRTC2 impacted cell growth in vitro (*SI Appendix,* Fig. S4*E*) and induced significant tumor stasis in vivo (Fig. 3*H* and *SI Appendix,* Fig. S4*F*), comparable to the tumor growth inhibition observed after reexpression of LKB1 (*SI Appendix,* Fig. S4*G*), demonstrating its key role in controlling tumor growth of established tumors.

**Fig. 4.**
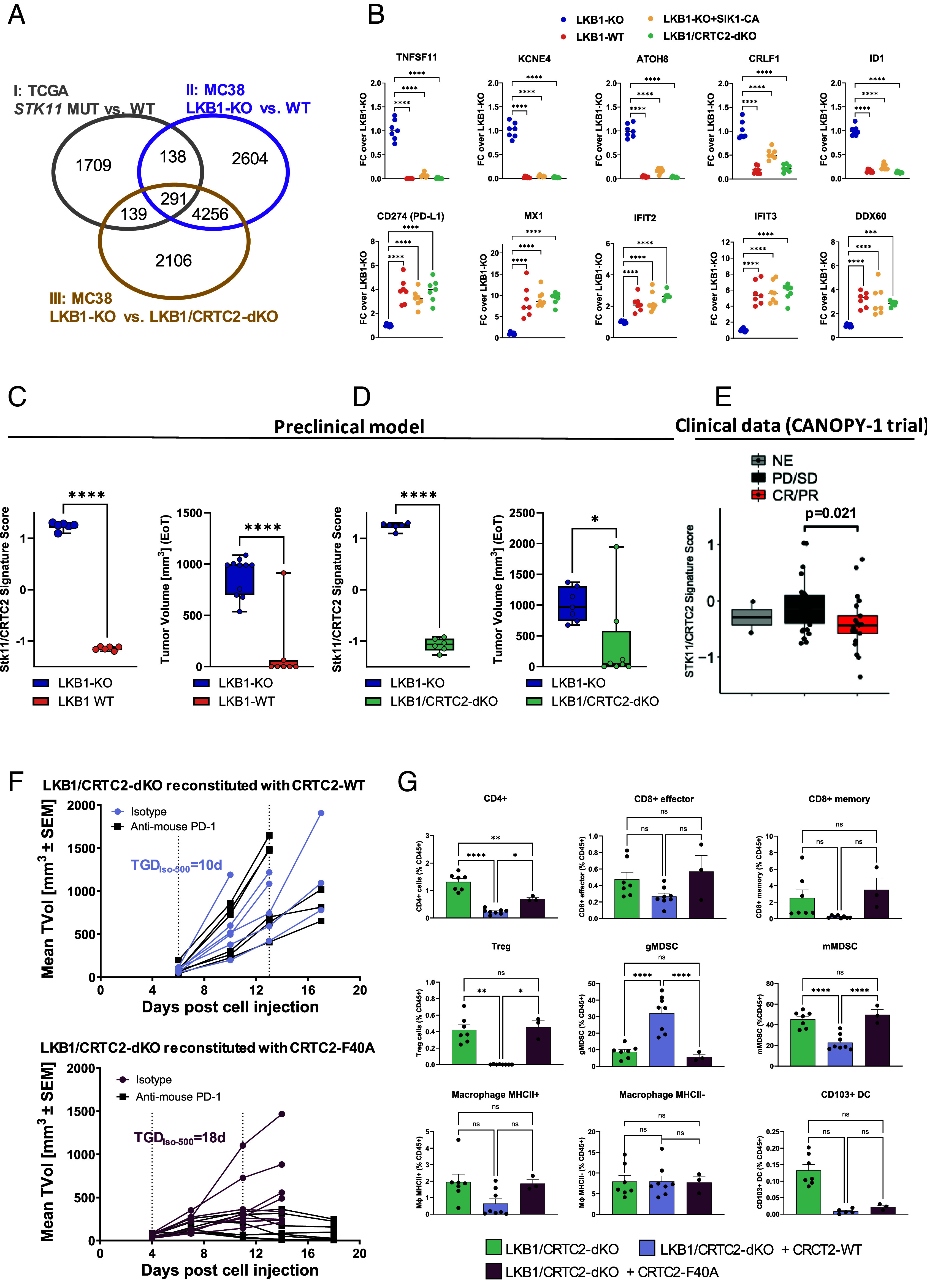
STK11/CRTC2 signature associates with response to immunotherapy and the abrogation of the binding between CRTC2 and CREB restores sensitivity to immunotherapy. (*A*) Venn diagram illustrates the overlap of differentially expressed genes (FDR < 0.01) among three datasets: *STK11* MUT vs. WT from TCGA (in gray), cancer cells isolated from LKB1-KO vs. WT MC38 tumors (in purple), and cancer cells isolated from LKB1-KO vs. LKB1/CRTC2-dKO MC38 tumors (in orange). (*B*) Scatter plots representing qRT-PCR for selected genes in cancer cells isolated from isotype-treated tumors presented in Fig. 3*D* and LKB1-WT tumors in Fig. 1*D*. Individual points represent different tumors, and the bar represents the median value. For normalization, the mean of the LKB1-KO group was set to 1. Statistical difference was calculated by one-way ANOVA with Dunnett’s multiple group comparison test. (*C* and *D*, *Left*) Boxplot illustrating the STK11/CRTC2 signature score derived from the intersection of the Venn diagram in LKB1-KO vs. WT MC38 tumors (*C*) and LKB1-KO vs. LKB1/CRTC2-dKO tumors (*D*), with the corresponding tumor volume (TVol) (*Right*) at the end of treatment (EoT) from experiment in Fig. 1*D* (*C*) and Fig. 3*D* (*D*). Statistical difference was assessed by the *t* test. (*E*) Boxplot illustrating the STK11/CRTC2 signature score of NSCLC tumors by patient Best Overall Response (BOR), defined as “Not Evaluable (NE),” “Progression Disease/Stable Disease (PD/SD),” and “Complete Response/Partial Response (CR/PR).” Statistical difference was calculated by the Mann–Whitney *U* test. (*F*) Tumor volumes (TVol) of MC38 cells with indicated genotypes and treated with either isotype control or anti-PD-1 antibody. Data are presented as individual tumor growth. Vertical dotted lines define the treatment time-points. TGD_Iso-500_ is the tumor growth delay to reach 500 mm^3^ for each individual tumor in the isotype treatment group. (*G*) Quantification of intratumoral immune populations from isotype-treated tumors with indicated genotypes (depicted as percentage of CD45^+^ cells). Data are presented as the mean ± SEM; statistical difference was assessed by using one-way ANOVA with Sidak’s multiple group comparison post hoc test.

To assess the translatability of our preclinical findings, we derived a STK11/CRTC2 signature shared among human and murine tumors (Fig. 4*A*). We calculated the differentially expressed gene (DEG) lists from the following comparisons: *STK11* mutant versus wild-type (WT) tumors from The Cancer Genome Atlas (TCGA), LKB1*-*KO versus WT tumors from the MC38 model, and LKB1*-*KO versus LKB1/CRTC2-dKO from the MC38 model (respectively I, II, and III in Fig. 4*A*). The 291 genes belonging to the STK11/CRTC2 signature arose from the intersection of these three DEG lists (Fig. 4*A* and *SI Appendix,* Table S1). Enrichment analysis of this signature revealed significant enrichment of pathways related to immune and inflammatory response, notably including the *Interferon Gamma Response*, *TNF-alpha Signaling via NF-kB* pathways and *Interferon Alpha Response,* in line with reported literature on *STK11* (5) (*SI Appendix,* Fig. S5*A*). A subset of these genes was validated, including both genes up- and down-regulated in a LKB1-null MC38 context, and SIK-CA and CRTC2-KO tumors showed a similar expression pattern to LKB1-proficient tumors (Fig. 4*B* and *SI Appendix,* Fig. S5*B*). In our preclinical models, this gene signature was high in LKB1-KO tumors and inversely associated with response to anti-PD-1 treatment (Fig. 4 *C* and *D*). We then analyzed RNA-Seq data from samples of nonsquamous NSCLC patients treated with the anti-PD-1 antibody pembrolizumab in combination with chemotherapy as a first-line treatment (control arm patients of the CANOPY-1 clinical trial NCT03631199) (29, 30). This signature was associated with *STK11* status (*SI Appendix,* Fig. S5*C*) and with the best overall response (BOR) to pembrolizumab in combination to chemotherapy (Fig. 4*E*). The STK11/CRTC2 signature showed a lower *P*-value than the STK11 signature (*SI Appendix,* Fig. S5*D*), suggesting that CRTC2 can further shape the gene expression program associated with positive response to ICB in *STK11* mutant tumors. Altogether, these results highlight CRTC2 as a potential node of intervention in this patient population. To functionally validate a targeting hypothesis for CRTC2, we evaluated if the disruption of the binding between CRTC2 and CREB was sufficient to resensitize LKB1-KO tumors to anti-PD-1 treatment. Indeed, CRTCs are the most potent coactivators of CREB but can also regulate other basic-region leucin zipper transcription factors, including c-Jun, c-Fos, and ATF-6 (31). CRTC2-deficient cells were reconstituted with either the wild-type or the F40A CREB-nonbinding mutant (32) isoforms of CRTC2 (*SI Appendix,* Fig. S5*E*). Only CRTC2-WT was able to rescue IL-11 production (at RNA and protein levels, respectively, *SI Appendix,* Fig. S5 *F* and *G*), increase aggressiveness of the tumors (Fig. 4*F*, isotype-treated tumor in the top graph compared to isotype-treated tumors in the bottom graph), abolish the response to anti-PD-1 treatment (Fig. 4*F*) and remodulate the tumor immune profile (Fig. 4*G*) similarly to LKB1-KO tumors, indicating that CRTC2-dependent phenotypes in the LKB1-deficient context are mediated by CRTC2–CREB protein interaction. Overall, these data shed light on CRTC2 as a key regulator of LKB1-dependent tumor immune-evasive functions and proposes the disruption of CRTC2–CREB interaction as a promising therapeutic approach for NSCLC *STK11* mutant tumors, currently recalcitrant to standard of care therapies and in urgent need for efficacious therapies.

## Discussion

Several studies have shown that *STK11* alterations in NSCLC are associated with primary resistance to ICB (2–4). Given the prevalence of *STK11* alterations and the lack of effective therapies for this patient population, understanding the underlying resistance mechanism and developing targeted approaches to overcome this resistance remain key priorities for the field. In this study, by utilizing isogenic LKB1 MC38 models, we demonstrated that loss of LKB1 causes resistance to anti-PD-1 treatment, in accordance with other reports using other preclinical models (3, 19, 33). A potential confounding factor could be the increased aggressiveness of LKB1-KO tumors (Fig. 1 *C*–*D*) (3, 32), as preclinical studies have shown that responsiveness to ICB often decreases as tumor aggressiveness increases. However, our data demonstrate that expressing the hypomorphic LKB1-K78I mutant in LKB1-KO tumors does not alter their growth rate, yet it fully restores sensitivity to anti–PD-1 therapy. This finding strengthens the conclusion that the impaired response of LKB1-KO tumors to anti–PD-1 is not primarily driven by differences in growth kinetics, but rather by the profound remodeling of the tumor immune microenvironment, including a conspicuous accumulation of gMDSCs, consistent with prior reports (3, 5, 19). Regarding the depletion of MDSC being sufficient to revert the resistance phenotype in a LKB1-KO background, discordant data have been reported. Selective depletion of gMDSC was not sufficient to resensitize CT26 model (3), while depletion of both gMDSC and mMDSC rendered KPL3 model sensitive to anti-PD-1 treatment (19). The discrepancy raised the question of whether the depletion of only gMDSC was insufficient for sensitization to anti-PD-1 treatment or if the findings were model-dependent. To clarify this, we assessed whether depleting both gMDSC and mMDSC with an anti-Gr-1 antibody could sensitize the MC38 model to anti-PD1 treatment. In our in vivo studies, depletion of MDSCs (although only partially sustained over time and therefore being an intrinsic limitation of the study) was not sufficient to sensitize LKB1-KO MC38 tumors to anti-PD-1 treatment (Fig. 1*F*), suggesting that the ability of MDSC deletion to revert resistance might be context-specific and not broadly generalizable. Of note, an additional possible confounding factor is that models where MDSC depletion did not resensitize are colorectal tumor models (CT26 and MC38).

Our research has identified SIK and CRTC2 as essential regulators of LKB1-mediated resistance to immune checkpoint blockade. While SIK1 and SIK3 have previously been recognized for their tumor suppressive roles downstream of LKB1 (10–12), their contribution to LKB1 immune functions was previously unknown. In the MC38 preclinical model, the expression of the constitutive form of SIK1 was sufficient to reverse the ICB resistance phenotype in LKB1-deficient tumors, without affecting tumor growth. This observation suggests redundancy among SIK family members in the regulation of tumor aggressiveness, in line with the literature (10, 11). In contrast, constitutive activation of each SIK isoform produced similar effects on the immunophenotype, indicating that this gain-of-function approach may exert a dominant effect over other family members. CRTC2 was found to regulate both cell-intrinsic and cell-extrinsic functions mediated by LKB1. Previous studies have shown that CRTC2 influences tumor initiation and establishment of *STK11* mutant tumors (12); however, its role in tumor maintenance remained unclear. We modeled therapeutic intervention by inducing knock-down of CRTC2 only after *STK11* mutant tumors were established and showed that CRTC2 depletion was sufficient to cause prolonged tumor stasis in multiple models. Notably, the impact of CRTC2 on tumor growth was restricted to the LKB1-deficient context in the MC38 model, emphasizing the need for further validation in additional NSCLC models. In addition, CRTC2 is a major regulator of LKB1-mediated transcriptional changes. More than 60% of LKB1-regulated genes are also regulated by CRTC2, including many immune-secreted factors, as previously shown in contexts other than cancer (34). Notably, CRTC2 can shape, together with LKB1, transcriptional programs associated with response to ICB in human tumors and its targeting was sufficient to remodel the TME of LKB1-KO tumors and sensitize them to anti-PD-1 therapy. Immunophenotypic profile revealed that low abundance of gMDSC, along with presence of mMDSC and CD4^+^ T cells, correlated with sensitivity to immunotherapy across all genetic perturbations examined. Importantly, we cannot exclude the possibility that other immune subpopulations not captured by our flow cytometry panel are associated with and potentially contribute to modulating the sensitivity to anti-PD-1 treatment.

Recently, other studies have linked STAT3 (3), STING (35, 36), Axl (37), ICAM1 (38), MCT4 (33), and CTLA4 (24) to ICB resistance of LKB1-deficient tumors. Each of the proposed mechanisms controls a specific part of the immune response, for example STING, Axl and ICAM regulate mainly T cell antitumor immunity, while STAT3 and MCT4 are primarily involved in myeloid cell biology and macrophage polarization. Interestingly, the expression of some of these proposed targets, such as STAT3, STING, and MCT4, is regulated by LKB1 and CRTC2 (see intersection of orange and purple circles in the Venn diagram of Fig. 4*A*), suggesting that CRTC2 could control multiple mechanisms, having a profound impact on the TME and resensitization to immunotherapy, in addition to a substantial effect on tumor growth. Importantly, the inhibition of the binding between CRTC2 and CREB was sufficient to restore sensitivity to anti-PD-1 treatment and impact the aggressiveness of LKB1-KO tumors, suggesting that this might be a possible path for a drug discovery approach. Since CRTC2-KO mice are viable and show only improved insulin sensitivity (39), we speculate that the selective targeting of CRTC2 might have a safe therapeutic index. On note, since CRTC2 plays a significant role in immune cell function, particularly in T cells (40), future studies are warranted to investigate the impact of CRTC2 inhibition in the tumor microenvironment. In conclusion, the findings in this manuscript suggest that CRTC2 is a promising target for *STK11* mutant NSCLC, having an impact both on tumor growth and response to immunotherapy.

## Material and Methods

### Cell Lines and Culture Conditions.

All cell lines were maintained in complete medium consisting of RPMI GlutaMAX supplemented with 10 mM HEPES, 1 mM sodium pyruvate, 1% MEM NEAA, 10% FBS, and 1% penicillin/streptomycin, and incubated at 37 °C with 5% CO_2_. A549, LL/2, and B16 cell lines were obtained from ATCC (as per Master Transfer Agreement between ATCC and Novartis Pharma AG). The CMT167 cell line was used as per a license agreement between Novartis Pharma AG and Cancer Research Technology Ltd (XIMBIO). The D4C9 cell line was used as per a license agreement with the Dana-Farber Cancer Institute (DFCI agreement #15066). The MC38 cell line was used as per agreements with the NCI/NIH (NCI MTA #38699-15; NIH Technology License #E-055-2016). All lines were tested for viral pathogens (IMPACT II, IDEXX BioAnalytics) and *Mycoplasma* contamination (*Mycoplasma* PCR detection kit, Sigma).

### Engineering of Isogenic Models.

KO models were generated by Cas9 RNP delivery using the Alt-R CRISPR-Cas9 system (IDT) and Neon electroporation device (Invitrogen). Briefly, predesigned target-specific crRNA (*SI Appendix,* Table S2) and tracrRNA were annealed at equimolar amounts and RNP complexes were formed by combining 3 μM of sgRNA with 2.5 μM of custom-made Cas9 recombinant protein. 4 × 10^5^ cells were electroporated (1,400 V, 30 ms, 1 pulse) with 10 μL tips and gene editing efficiency was assessed by immunoblot after 5 d. Clones were subsequently derived by serial dilutions, selected by CloneSelect Imager (Molecular Devices), and confirmed by immunoblot. Transgenic cell lines were generated by lentiviral spin-infection (400 g, 90 min, 37 °C) in polybrene-containing medium (8 μg/mL). 5 × 10^4^ cells were plated in a 24-well plate and transduced the next day with various amounts of virus to ensure a MOI < 1.

### Lentiviral Expression Vectors and Lentiviral Production.

All constructs used were produced by gene synthesis of the codon-optimized cDNA (*SI Appendix,* Table S3) into a custom lentiviral vector under an EF1a promoter; for the CRTC2-WT rescue construct the CRTC2 native promoter was used. Constitutive-active substrates of LKB1 were generated by mutating the threonine residue in the T-loop site into glutamic acid. Doxycycline-inducible shCRTC2 constructs were purchased from Dharmacon (#V3SH11252-225731363 for A549 cells and V3SH11252-228079544 for H2122 cells). Lentiviral particles were produced by cotransfection of 293FT cells (ThermoFisher) with the pC-Pack2 packaging plasmid (Cellecta) using the TransIT transfection reagent (Mirus) at a DNA:TransIT ratio of 1:3. The cell medium was refreshed 24 h posttransfection and the viral supernatant collected 48 h later. Selection of transduced cells was performed with blasticidin (10 μg/mL) and puromycin (1 μg/mL).

### In Vivo Studies.

All animal studies were conducted in accordance with ethics and procedures covered by permit BS-1975 and BS-2921 issued by the Kantonales Veterinäramt Basel-Stadt and in strict adherence to guidelines of the Eidgenössisches Tierschutzgesetz and the Eidgenössische Tierschutzverordnung, Switzerland. All animal studies were approved by the internal ethics committee. All animals had access to food and water ad libitum and were identified with transponders. They were housed in a specific pathogen-free facility in IVC racks with a 12 h light/12 h dark cycle. To establish mouse MC38-derived xenograft models, 6 to 7-week-old female C57/Bl6N mice from Taconics or athymic nude mice (Crl:NU(NCr)-Foxn1nu) mice from Charles River were engrafted subcutaneously with 1 million cells in HBSS (#H6648, Sigma). For human cell line-derived xenograft models, 6 to 7-week-old female athymic nude (Crl:NU(NCr)-Foxn1nu) mice from Charles River were engrafted subcutaneously with 5 million cells in HBSS (#H6648, Sigma). For H2122, the cells were concentrated in 50% Matrigel (#354234, Corning). The mouse PD-1 inhibitor 1D2 was dissolved in PBS. Doxycycline hyclate (#D9891, Sigma-Aldrich) was dissolved in 10% Sucrose (#S0389, Sigma-Aldrich) at a concentration of 2 mg/mL and added to drinking water. The water bottle was changed twice a week. For efficacy experiments in MC38 models, mice were randomized into groups (n = 8) for a mean tumor size of 50 mm^3^ and dosed with either isotype or 1D2 at 10 mg/kg i.v. once weekly for 2 to 4 wk. Tumor aggressiveness of a model is estimated by calculating the time to reach 500 mm^3^ for each individual tumor in the isotype treatment group (Tumor Growth Delay or TGD_Iso-500_). For selective elimination of immune cell populations in C57BL/6 mice with MC38 tumors, the mice were treated, starting 3 d after the cell injection, with 200 µg anti-Gr-1 (BioXcell, Clone RB6-8C5 #BE0075) i.p. every 2 d or isotype control (BioXcell, Clone LFT-2 #BE0090). The mouse PD-1 inhibitor 1D2 (or isotype) was administered at 10 mg/kg i.v. once weekly. At the end of the efficacy experiment, tumors were collected for immune-phenotyping analysis. For efficacy experiment in A549 and H2122 models, mice were randomized into groups (n = 6 to 8) for a mean tumor size of 100 mm^3^ and dosed with either sucrose or doxycycline for 3 to 4 wk. Tumor responses are reported with the measures of tumor volumes (TVol) from the treatment start. It is quantified either by the change in TVol (endpoint minus starting value in mm^3^) as the T/C or by the percentage of regression of the starting TVol. Statistical differences between the means of TVol were assessed on the endpoint ∆Tvol using an unpaired *t* test with *P* < 0.05 considered as significant.

### Dissociation of In Vivo Tumors.

Freshly isolated MC38 tumors were cut into pieces and homogenized to single cells by enzymatic treatment with the mouse tumor dissociation kit (Miltenyi) on a gentle MACS tissue dissociator system (Miltenyi) at 37 °C for 40 min. Enzymatic digestion was stopped by dilution with cold PBS containing 2% FBS and tumor cells pelleted by centrifugation at 400 g for 5 min at 4 °C. Following lysis of red blood cells with ACK buffer (Gibco), cells were diluted in PBS-FBS 2% and filtered through a 70 μm strainer before counting with a TC20 counter (BioRad) for further use.

### Flow Cytometry Analysis.

0.3 to 1 × 10^6^ cells collected from dissociated MC38 tumors were labeled with the viability dye FVS450 (BD Horizon) diluted in PBS-FBS 2% for 30 min at 4 °C, washed, and preincubated with the TruStain FcX antibody (BioLegend) for 15 min to block Fc receptors. Cells were then pelleted and stained for surface marker expression with a MDSC-focused antibody panel in the MDSC depletion study (*SI Appendix,* Table S4) or a broad antibody panel for immunophenotyping purpose (*SI Appendix,* Table S5). After 30 min incubation at 4 °C of the antibody cocktails diluted in brilliant stain buffer (BD Horizon), cells were washed and further fixed and permeabilized with the transcription factor buffer set (BD Pharmingen) for intracellular staining. Ly6G and FoxP3 antibodies were incubated for 30 min at 4 °C in the MDSC depletion experiments (22) and immunophenotyping studies respectively followed by cell washes. Flow cytometry analysis was performed on a CytoFLEX instrument (Beckman) using the gating strategies described in *SI Appendix,* Fig. S1*C* and quantification achieved with FlowJo (BD Biosciences).

### MDSC Sorting and Cellular Assays.

Cells collected upon dissociation of isogenic LKB1 tumors were labeled with the viability dye FS450 (BD Horizon), preincubated with the TruStain FcX antibody (BioLegend) and stained with the antibody panel listed in *SI Appendix,* Table S6. Upon sorting with a FACSAria instrument (BD Biosciences), gMDSC and mMDSC populations were tested in T cell suppression and migration assays.

T cells freshly isolated from the mouse spleen (pan T cell isolation kit II, Miltenyi) were labeled with Tag-it violet (BioLegend), seeded at 50 × 10^4^ cells/well and cocultured with MDSC at different ratios (T cell: MDSC ratios 32:1, 16:1, 8:1, 4:1&2:1). IL-2 (10 ng/mL, Peprotech), IL-6 (40 ng/mL, Miltenyi), GM-CSF (40 ng/mL, Miltenyi), and dynabeads (Miltenyi, T-cells/ beads ratio 1:2) were added and cells were cultured for 4 d before flow cytometry analysis was performed.

Recruitment of MDSC by tumor cells was assessed by transwell migration through 3 μm pore size inserts (Millipore, #PITP01250). Briefly, conditioned media from isogenic cells were produced by growing 5 × 10^5^ cells/well in 2 mL of 0.5% FCS-containing medium in 6-well plates for 48 h. 2 × 10^5^ freshly sorted MDSC diluted in 200 μL medium-FCS 0.5% were added to the top chamber of the transwell plate and migrated toward 400 μL of conditioned media in the bottom chamber. Cell migration was measured after 90 min by luminescence with CellTiter-Glo reagent (Promega) using an Infinite M200 reader (Tecan) and the absolute cell number determined by comparison with MDSC-generated standard curves.

### ELISA for IL-11.

IL-11 production was measured with the mouse SimpleStep kit (Abcam, #ab215084) according to manufacturer protocol. Conditioned media were produced by seeding 2 × 10^5^ c/w in 2 mL of complete medium in 12-well plate for 48 h and tested undiluted. Sera were diluted 1:1 with diluent buffer.

### Immunoblot.

Cell lysates for the detection of total protein amounts were prepared in RIPA buffer (Pierce) for 30min on ice, centrifuged at 4 °C and boiled in reducing conditions with Laemmli buffer (BioRad). Cell lysates for the detection of phosphorylated proteins were prepared rapidly by digesting gDNA with benzonase for 5min (50 U/mL, Merck) in a benzonase-compatible lysis buffer (RIPA diluted 1:1 with a Tris-HCl 25 mM, NaCl 150 mM solution) and boiling samples with no centrifugation step. Lysis buffers were supplemented with Halt protease and phosphatase inhibitors (Pierce) and lysates normalized with a BCA assay kit (Pierce). Proteins were resolved on 4 to 12% or 6% SDS-PAGE gels, transferred onto PVDF membranes (Thermo Scientific) and probed with the following antibodies: LKB1 (CST, #3047), CRTC1 (CST, #2587), CRTC2 (Millipore, # MABN695 for mouse samples and Abcam, # ab109081 for human samples), CRTC3 (CST, #2720), pan-pSIK (Abcam, # ab199474), and vinculin (CST, #13901).

### Soft Agar Colony Formation Assay.

2.5 × 10^4^ cells were diluted in complete medium containing 0.35% agarose (Thermo Scientific, #17856) and plated on top of a polymerized layer of 0.8% agarose in 6-well plates. Medium containing doxycycline at concentration 100 ng/mL and 25 ng/mL for A549 and H2122 cells respectively was refreshed twice a week. Colonies were stained after 3 wk with 0.004% crystal violet (Sigma) for 1 h, imaged with an Odyssey system (LI-COR) and quantified using Fiji (41).

### qRT-PCR of Tumor Cells.

Cancer cells from dissociated tumors collected at the end of the in vivo studies were isolated by negative selection with CD45 microbeads using a MACS system (Miltenyi) according to manufacturer protocol. RNA was extracted from tumor cell pellets with a QIAshredder/RNeasy Plus Mini kit (Qiagen) and cDNA synthesized with a QuantiTect Reverse Transcription kit (Qiagen). SYBR Green qPCR (QuantiNova, Qiagen) was performed on an ABI 7900HT instrument with predesigned and custom primer sets (IDT) listed in *SI Appendix,* Table S7.

### Mouse Tumor RNA Sequencing.

Dissociated tumors and in vitro cell cultures were lysed using Promega© RNA-Seq Direct Cell Lysis System (CS318801) to produce total RNA lysates that are stable, DNA-free, and ready for sequencing library preparation without the need for dilution or further purification. The resulting lysate solution was used as input for Illumina’s TruSeq Stranded mRNA Library Prep Kit, High Throughput (RS-122-2103). Sample libraries were generated per manufacturer’s specifications using an automation method developed on the Hamilton STAR robotics platform. The PCR amplified RNA-Seq library products were then quantified using the Agilent Technologies TapeStation 4200 D1000 kit (5067-5583). The samples were diluted to 10 nanomolar in Qiagen Elution Buffer (Qiagen, 1014609), denatured, and loaded at a range of 2.5 to 4.0 picomolar on an Illumina cBOT using the HiSeq® 4000 PE Cluster Kit (PE-410-1001). The RNA-Seq libraries were sequenced on a HiSeq® 4000 at 75 base pair paired end with eight base pair dual indexes using the HiSeq® 4000 SBS Kit, 150 cycles (FC-410-1002). The sequence intensity files were generated on instrument using the Illumina Real Time Analysis software. The resulting intensity files were demultiplexed with program bcl2fastq2 and aligned to the human transcriptome using the open-source pipeline PISCES (42). FASTQ files are available on the SRA repository under accession number PRJNA1445085 (PRJNA1445085 - SRA - NCBI).

### Mouse Tumor RNA-Sequencing Data Processing.

Transcript-level quantification was performed using PISCES and the Ensembl GRCm38 Release 13 reference mouse genome. Raw counts and transcripts per Million (TPM) were calculated and used for analysis. The EdgeR package was used for Trimmed Mean of M-values (TMM) normalization and differential gene expression analysis (41). Gene signature scores were calculated by taking the average of Logarithm of 2 scaled TPM counts for a set of genes for each sample.

### CANOPY-1 Biomarker Cohort.

CANOPY-1 study (NCT03631199) was a phase III, randomized, double-blind, global study evaluating the efficacy and safety of pembrolizumab plus platinum-based doublet chemotherapy combined with either canakinumab or placebo in NSCLC as first-line therapy with published clinical results and biomarker results (29, 30). The study was conducted in accordance with the principles of the Declaration of Helsinki and the Good Clinical Practice guidelines of the International Council for Harmonisation. Control-arm patients with written informed consent and nonsquamous histology were included for the analysis to validate LKB1 expression signature. Specifically, for 57 nonsquamous patients in the control-arm there were available RNA-Seq data from baseline tumor biopsies using in-house RNAse H-based assay (43) and tumor mutation data profiled by FoundationOne CDx assay (Foundation Medicine).

### TCGA LUAD Data Processing.

TCGA LUAD RNA-Seq data were deconvoluted by BayesPrism (44) using cell-by-gene raw count matrix of NSCLC single-cell RNA-seq data (45) as the reference data. The deconvolution performance reached a median Pearson’s correlation of 0.90 between deconvoluted tumor epithelial cell gene expression with ground-truth epithelial gene expression directly measured in original single-cell data (46). Deconvoluted TCGA LUAD tumor counts were further normalized using TPM. TCGA LUAD STK11 alteration was retrieved from cbioportal (47) where STK11mut was defined for samples with either functional missense/nonsense/frameshift variants or deletion.

## Supplementary Material

Appendix 01 (PDF)

## Data Availability

RNA-seq FASTQ files data have been deposited in SRA repository [Accession number: 432 PRJNA1445085–SRA–NCBI)] (48). Some study data are available. Data related to clinical signature can be shared upon request to the corresponding author.
