# Supplementary material for: Targeting CRTC2 reverses STK11 mutant NSCLC tumor resistance to immunotherapy
Source: Proc Natl Acad Sci U S A. 2026 Apr 22;123(17):e2508762123. doi: 10.1073/pnas.2508762123 (PMC13123801; doi:10.1073/pnas.2508762123)

Supplementary Figure 1 Impact of LKB1 on intratumoral MDSC. (A-B) Western blot analysis of **(A)** single cell MC38 clones derived from transient expression of sg*STK11* and **(B)** LKB1-KO clone B11 cells transduced with different constructs. Lysates from cells were prepared and proteins detected with the indicated antibodies. **(C)** Schematic of the gating strategy utilized to characterize intratumoral immune populations by Flow Cytometry. **(D)** Representative image of Flow Cytometry plot of intratumoral MDSC cells from LKB1-KO (*left*) and LKB1-WT (*right*) MC38 tumors. Dark and light blue rectangles identify respectively gMDSC and mMDSC from LKB1-KO tumors, and pink rectangle identifies mMDSC from LKB1-WT tumors. **(E)** mMDSC and gMDSC were isolated from tumors with indicated genotypes and then cultured with CD3/CD28-stimulated T cells isolated from the spleen of non-tumor bearing mice to induceT-cell proliferation *ex-vivo*. E:T ratios indicate the MDSC:T-cell ratio in co-culture at fixed T-cell numbers. Data are normalized to T cell alone control and set to 100%. **(F)** mMDSC and gMDSC were isolated from tumors with indicated genotypes (dark and light blue respectively for gMDSC and mMDSC from LKB1-KO tumors, pink for mMDSC from LKB1-WT tumors) and their ability to be recruited by cancer cells was assessed with a transwell assay. x-axis groups data according to the genotype of the cancer cells utilized to produce the supernatants (SUP) for the transwell assay (cell culture medium was employed as control). Statistical difference was calculated by one-way ANOVA with Sidak's multiple group comparison test**. (G-I)** Quantification of intratumoral gMDSC **(G)** and mMDSC **(H)** cells 4 days after treatment. **(I)** Quantification of intratumoral mMDSC cells from tumors in Fig. 1F 9 days after treatment. Data are presented as the mean±SEM and depicted as percentage of live cells; statistical difference was calculated by using one-way ANOVA with Sidak's multiple group comparison test.

Supplementary Figure 2 LKB1-associated transcriptomic changes in LKB1 murine isogenic pairs models. (**A-F**) MA plots showing the comparisons of: LKB1-KO vs. LKB1-WT CMT167 cell line (**A**), LKB1-KO vs. LKB1-WT B16 cell line (**B**), LKB1-KO vs. LKB1-WT D4C9 cell line (**C**), LKB1-KO vs. LKB1-WT MC38 cell line (**D**), LKB1-KO vs. LKB1-K78I MC38 cell line (**E**), LKB1-KO vs. LKB1-D194A MC38 cell line (**F**). (**G**) Heatmap of RNA expression levels (as measured by RNA-seq) of genes highlighted in Fig. 2A and B. (**H**) IL-11 levels were quantified by ELISA from the sera of isotype-treated mice bearing LKB1-KO and LKB1-WT tumors presented in Fig. 1D. Data are presented as the mean±SEM. Statistical difference was calculated by t-test.

Supplementary Figure 3 CRTC2 regulates LKB1-dependent functions. (A) Bar plot depicting the exogenous expression levels of the cDNA constructs expressed in MC38 cells indicated in the x-axis measured by qRT-PCR. **(B)** Western blot analysis of cells with indicated genotypes. Lysates from cells were prepared and proteins detected with the indicated antibodies. **(C)** *(Top)* IL-11 levels were quantified by ELISA from the supernatants of the MC38 cells with indicated genotypes. For normalization, the LKB1-KO value of each experiment was set to 100%. Data are presented as the mean±SEM. Statistical difference was calculated by one-way ANOVA with Dunnett's multiple group comparison test. *(Bottom)* Western blot analysis of cells with indicated genotypes. Lysates from cells were prepared and proteins detected with the indicated antibodies. **(D)** Western blot analysis of cells with indicated genotypes. Lysates from cells were prepared and proteins detected with the indicated antibodies. EV=empty vector. **(E)** IL-11 levels were quantified by ELISA from the supernatants of cells with indicated genotypes. For normalization, the LKB1-KO value of each experiment was set to 100%. Data are presented as the mean±SEM. Statistical difference was assessed with a one-way ANOVA with Dunntett's multiple group comparison post-hoc test. **(F)** Schematic cartoon of CRTC2 regulation in LKB1 proficient and deficient cells. **(G)** Western blot analysis of cells with indicated genotypes. Lysates from cells were prepared and proteins detected with the indicated antibodies. LKB1 was expressed constitutively in MC38 cells and in a doxycyclin-inducible manner (dox) in A549 cells.

Supplementary Figure 4. **CRTC2 role in tumor maintenance. (A)** Quantification of intratumoral immune populations from tumors with indicated genotypes (depicted as percentage of $CD45^+$ cells). Data are presented as the mean± SEM; statistical difference was assessed by using one-way ANOVA with Sidak's multiple group comparison post-hoc test. **(B)** Western blot analysis of cells with indicated genotypes. Lysates from cells were prepared and proteins detected with the indicated antibodies. **(C)**

Violin plot representing the time for MC38 tumors with indicated genotypes to reach 500 mm^3. Statistical difference was assessed with a one-way ANOVA with Sidak's multiple group comparison test. **(D)** Western blot analysis of cells transduced with inducible shRNA against a non-targeting control (Ctrl) or CRTC2 and treated or not with doxycycline (100ng/ml for A549 and 25ng/ml for H2122) for 72 hrs. Lysates were prepared and proteins detected with the indicated antibodies. **(E)** Cells with indicated genotypes were seeded for soft agar colony formation assay and treated or not with doxycycline (100ng/ml for A549 and 25ng/ml for H2122). Colonies were counted at the end of the experiment and data are presented as the mean$\pm$SEM. p value was calculated by one-way ANOVA with Sidak's multiple group comparison test. **(F)** After tumors from A549 (*left*) and H2122 (*right*) cells transduced with an inducible shRNA against a non-targeting control reached 100 mm^3 size, mice were randomized and treated with 10% sucrose or doxycycline. Tumor volumes (TVol) are presented as the mean$\pm$SEM. **(G)** After tumors from A549 cells transduced with an inducible LKB1 construct reached 100 mm^3 size, mice were randomized and treated with 10% sucrose or doxycycline. Tumor volumes (TVol) are presented as the mean$\pm$SEM. Statistical differences at the end of treatment were assessed by using an unpaired t-test.

Supplementary Figure 5 CREB interaction is essential for CRTC2 activity. (A) Five top pathways from enrichment analysis of STK11/CRTC2 signature using EnrichR against the 2020 MSigDB Hallmark collection **(B)** Scatter plots representing qRT-PCR for selected genes in cancer cells isolated from tumors presented in Supplementary Fig. 4A. Individual points represent different tumors, and the bar represents the median value. For normalization, the mean of the LKB1-KO group was set to 1. Statistical difference was assessed by using one-way ANOVA with Sidak's multiple group comparison post-hoc test. **(C)** Boxplot illustrating the STK11/CRTC2 gene signature score in NSCLC samples from CANOPY-1 separated by *STK11* alteration status. Statistical difference was calculated using Mann-Whitney U test. **(D)** Boxplot illustrating the STK11 signature score of NSCLC tumors by patient Best Overall Response (BOR), defined as 'Not Evaluable (NE)', 'Progression Disease / Stable Disease (PD/SD)', and 'Complete Response / Partial Response (CR/PR)'. Statistical difference was calculated by Mann-Whitney U test. **(E)** Western blot analysis of cells lines with indicated genotypes. Lysates from cells were prepared and proteins detected with the indicated antibodies. **(F-G)** IL-11 levels were quantified by qRT-PCR **(F)** and ELISA **(G)** from MC38 cells with indicated genotypes. EV=empty vector. For normalization, the LKB1-KO value of each experiment was set to 100%. Data are presented as the mean$\pm$SEM. Statistical difference was calculated by one-way ANOVA with Sidak's multiple group comparison test.

Supplementary Table 1: *STK11/CRTC2* signature

Gene name	Gene name	Gene name	Gene name	Gene name	Gene name
ACADM	CLIC4	GAK	LIMK1	PRKCA	SPIRE2
ACKR3	CNPY2	GALNT2	LNPEP	PRMT8	SPRY2
ACSL3	COG2	GBP6	LPAR1	PROSER2	STEAP3
ADD3	COPA	GCH1	LPCAT2	PRPF40B	SYBU
ADGRG3	COPZ1	GCNT2	LRPAP1	PRUNE1	TAOK3
ADGRV1	COX4I2	GEM	LRRN3	PTAR1	TBC1D2
ADH7	CRABP2	GHR	LTBR	PTDSS2	TBC1D8
ADSSL1	CRACR2A	GLMP	LYSMD1	PTGES	TCF25
AIFM2	CRLF1	GLS	MARC2	PTOV1	TEX9
ALDH3A1	CSF3	GMPPB	MCC	PTPRN	TFRC
ALDH3B1	CST3	GPAA1	MEF2C	RAB23	THPO
ANO6	CTBP2	GPR39	MET	RASAL2	TMED3
ANO9	CYHR1	GPRC5B	MFSD3	REEP6	TMEM176A
AQP1	CYTH3	GPSM2	MFSD9	RELN	TMEM176B

ARHGAP10	DCXR	GRAMD1A	MIA3	REV3L	TMEM59L
ARMC8	DDX60	GRINA	MME	RFK	TNFRSF1A
ARRB1	DECR1	GXYLT2	MOB3B	RGL1	TNFSF11
ATG101	DECR2	HERC3	MORC3	RGS2	TOMM6
ATOH8	DENND4C	HERC6	MRPS6	RHOBTB1	TRABD
ATP10A	DESI1	HES6	MRS2	RHOU	TRAPPC9
AVPI1	DGAT1	HMGA2	MTM1	RNF145	TSPAN11
B4GALNT4	DNAJC1	HOXA9	MTMR7	RPN2	TSPAN5
B4GALT1	DNAJC10	HPN	MUC1	RPS6KA3	TSTA3
B4GALT3	DNAJC12	ID1	MX1	RRAGC	TTC39C
BAALC	DSTN	ID2	MXRA7	RRBP1	TXNIP
BICD1	DUSP16	ID3	NAXD	RSAD2	UCK2
BMP2	EBF3	IFFO2	NEDD9	RSPO3	UFC1
BRINP3	EBPL	IFI44	NF2	S100A10	UPP1
BTG2	ECHS1	IFIT1	NFIX	SAMD9L	VIM
C3	ECI2	IFIT2	NR1D2	SAMHD1	VPS37A
CACHD1	EDEM2	IFIT3	NR4A1	SCCPDH	WDFY4
CACNB3	EFNA1	IGSF8	NR4A2	SDF2L1	WIF1
CAMK2D	EMC10	IL1RAP	NSMF	SDK1	WNT10B
CCDC170	EML4	IMPA2	NUDT4	SEC16A	XAF1
CCL2	EMP3	IRS2	OAT	SEPT11	XBP1
CCL7	ENO3	ITGA6	OPLAH	SERPINF1	YPEL2
CD274	EPHA7	JAG2	OS9	SESN2	ZBED3
CD99L2	EPS15	JUP	PAQR3	SH3BP4	ZBTB42
CDC14A	ERGIC1	KAT2B	PBXIP1	SIAE	ZEB2
CDSN	ERGIC3	KCNE4	PCYOX1	SIK1	ZMYM1
CEBPE	EVI2A	KCNN4	PDE4D	SIL1	ZNFX1
CEP57L1	FARS2	KIF1C	PDIA3	SLC20A2	
CERK	FIGNL1	KLK11	PHLDB1	SLC25A11	
CFLAR	FKBP10	KRT16	PIGO	SLC30A4	
CHID1	FOXA1	KSR1	PIGT	SLC39A7	
CHMP1B	FRS3	LAMTOR5	PIK3CB	SLC4A7	
CHP1	FRYL	LAYN	PIM3	SLC5A3	
CHPT1	FZD9	LGALS1	PITPNC1	SLPI	
CISD1	G6PC3	LHFPL2	PLSCR4	SOCS2	
CLCA2	GADD45A	LIMA1	PRDX6	SPATS2	

Supplementary Table 2 List of CRISPR RNA used for Cas9 gene editing

Gene	crRNA IDT ID	crRNA sequence
STK11	Mm.Cas9.STK11.1.AC	TTGACGTTGGCCTCTCCATT
CRTC1	Mm.Cas9.CRTC1.1.AA	GTCGGACCACACGACATCAT
CRTC2	Mm.Cas9.CRTC2.1.AA	CTCGGAGCACTCGGCACCAT
CRTC3	Mm.Cas9.CRTC3.1.AA	GCGGGTTCCACGAACATTAT
OlfR571	custom	GTACATTGGTCGATGCAATG

Supplementary Table 3 cDNA constructs

Construct	Sequence ID	Mutation
LKB1-WT	NP_035622.1	-
LKB1-K78I	NP_035622.1	K78I
LKB1-D194A	NP_035622.1	D194A
BRSK1	NP_001003920.2	T189E
BRSK2	NP_001009930.1	T175E
NUAK1	NP_001004363.1	T212E
NUAK2	AAH33302.1	T212E
MARK1	NP_001365805.1	T215E
MARK2	NP_031954.2	T208E
MARK3	NP_001357673.1	T211E
MARK4	NP_758483.1	T214E
SIK1	NP_034961.2	T182E
SIK2	NP_848825.2	T175E
SIK3	NP_081774.3	T221E
AMPKa1	NP_001013385.3	T183E
AMPKa2	AAI38567.1	T172E
CRTC2-WT	NP_083157.1	-
CRTC2-F40A	NP_083157.1	F40A

Supplementary Table 4 Antibody panel for MDSC depletion analysis

FACS Antibody	Provider	Cat #
PerCP-Cy5.5 Rat Anti-Mouse CD45	BD	550994
BUV661 Rat Anti-CD11b	BD	612977
APC/Cyanine7 anti-mouse Ly-6C	BioLegend	128026
BV510 anti-mouse Ly-6G	BioLegend	127633
FITC anti-mouse Ly-6G	BioLegend	127606
PerCP-Cy5.5 Rat IgG2b, κ Isotype	BD	550764
BUV661 Rat IgG2b, κ Isotype	BD	612978
APC/Cyanine7 Rat IgG2c, κ Isotype	BioLegend	400719
BV510 Rat IgG2a, κ Isotype	BioLegend	400553
FITC Rat IgG2a, κ Isotype	BioLegend	400505

Supplementary Table 5 Antibody panel for immunophenotyping analysis

Antibody	Provider	Cat #
PerCP-Cy5.5 Rat Anti-Mouse CD45	BD	550994
BUV661 Rat Anti-CD11b	BD	612977
BUV395 Rat Anti-Mouse CD4	BD	563790
Super Bright 780 Rat Anti-Mouse CD8a	ThermoFisher	78-0081-82
APC Rat Anti-FoxP3	ThermoFisher	17-5773-82
BV605 Hamster Anti-Mouse CD11c	BD	563057
PE anti-mouse CD103	BioLegend	121406
APC/Cyanine7 anti-mouse Ly-6C	BioLegend	128026
BV510 anti-mouse Ly-6G	BioLegend	127633
BV650 anti-mouse F4/80	BioLegend	123149
Alexa Fluor 700 anti-mouse MHCII	BioLegend	107622
PerCP-Cy5.5 Rat IgG2b, κ Isotype	BD	550764
BUV661 Rat IgG2b, κ Isotype	BD	612978
BUV395 Rat IgG2a, κ Isotype	BD	563556
Super Bright 780 Rat IgG2a, κ Isotype	ThermoFisher	78-4321-80
APC Rat IgG2b, κ Isotype	BioLegend	400612
BV605 Hamster IgG1, λ1 Isotype	BD	563054
PE Armenian Hamster IgG Isotype	BioLegend	400908
APC/Cyanine7 Rat IgG2c, κ Isotype	BioLegend	400719
BV510 Rat IgG2a, κ Isotype	BioLegend	400553
BV650 Rat IgG2a, κ Isotype	BioLegend	400542
Alexa Fluor 700 Rat IgG2b, κ Isotype	ThermoFisher	56-4031-80

Supplementary Table 6 Antibody panel for sorting MDSC

Antibody	Provider	Cat #
PerCP-Cy5.5 Rat Anti-Mouse CD45	BD	550994
PE Rat Anti-CD11b	BioLegend	101208
APC/Cyanine7 anti-mouse Ly-6C	BioLegend	128026
BV510 anti-mouse Ly-6G	BioLegend	127633

Supplementary Table 7 List of primers used for qRT-PCR analysis

Primer sets with the indicated assay ID below were predesigned by IDT. Custom primer sets were designed with PrimerQuest Tool from IDT.

Gene	Provider	Assay ID	PRIMER SEQUENCE	
IL11	IDT	Mm.PT.58.9688576	Fwd	GGA TCA CAG GTT GGT CTG G

			Rev	CGA CTG GAA CGG CTA CTC
CXCL5	IDT	Mm.PT.58.29518961.g	Fwd<br>Rev	ATC ACC TCC AAA TTA GCG ATC A<br>TTC TGT TGC TGT TCA CGC T
CXCL10	IDT	Mm.PT.58.43575827	Fwd<br>Rev	TGA TTT CAA GCT TCC CTA TGG C<br>ATT TTC TGC CTC ATC CTG CT
CXCL11	IDT	Mm.PT.58.42838989	Fwd<br>Rev	GGG CCG ATG CAA AGA CA<br>GAG ATG AAC AGG AAG GTC ACA G
TNFSF11	IDT	Mm.PT.58.29202697	Fwd<br>Rev	AGT GCT GTC TTC TGA TAT TCT GT<br>TCC CGC TCC ATG TTC CT
CRLF1	IDT	Mm.PT.58.10244905	Fwd<br>Rev	GTG TGT CTC CAT GTA TAG AGC AG<br>CTC TGT TGC TCT GTG TCC TC
ATOH8	IDT	Mm.PT.58.9189606	Fwd<br>Rev	GCT TTC TCC TCA GTC CCA AC<br>CTC AGC TTC TCC GAG TGT G
ID1	IDT	Mm.PT.58.6622645.g	Fwd<br>Rev	CAG CGA CAC AAG ATG CGA T<br>CTG AAC TCG GAG TCT GAA GTC
KCNE4	IDT	Mm.PT.58.8176180	Fwd<br>Rev	CCA TCC TCA GCA TTG AAT TGC<br>CCG TTT AAG AGT TCC ACA CTT G
CD274	IDT	Mm.PT.58.11921659	Fwd<br>Rev	CCA CAT TTC TCC ACA TCT AGC A<br>TCC ATC CTG TTG TTC CTC ATT G
DDX60	IDT	Mm.PT.58.6179282	Fwd<br>Rev	ATG GTA CTG TCT ACT TGC TTC C<br>CTC CTT GTT ATG ATC CGA TGT CC
MX1	IDT	Mm.PT.58.12101853.g	Fwd<br>Rev	GGT GTC GAT GAG GTC AAT ACA G<br>ACC AAG TAA ACA TCC TGA TAC CTC
IFIT2	IDT	Mm.PT.58.28800045.g	Fwd<br>Rev	GAG TCT ACT GCT TTG CAC AGA T<br>GTT CAA CTG TTC CAT TAG CTC AG
IFIT3	IDT	Mm.PT.58.33537107	Fwd<br>Rev	CTT CAG CTG TGG AAG GAT CG<br>CAC ACC CAG CTT TTC CCA
GAPDH	IDT	Mm.PT.39a.1	Fwd<br>Rev	GTG GAG TCA TAC TGG AAC ATG TAG<br>AAT GGT GAA GGT CGG TGT G
BRSK1	IDT	Custom primer set	Fwd<br>Rev	CAA ACC TCT GTC CTC CAT CAA<br>GGG TCC TCC TGA AGC TTT ATA C
BRSK2	IDT	Custom primer set	Fwd<br>Rev	CTG CTG GAT GAG CGG AAT AA<br>ATG ACT TCG GGA CAA GCA TAG
NUAK1	IDT	Custom primer set	Fwd<br>Rev	AAC AGA GGG AGA GTG GGT ATT A<br>GAT GAT AGA CCG CCG GAA ATT A
NUAK2	IDT	Custom primer set	Fwd<br>Rev	GCG CGA GTC CGG TTA TTA TAG<br>CAG GAT CGC CAC TAA CGA ATA C
MARK1	IDT	Custom primer set	Fwd<br>Rev	CCC TCT ATC TGG TCA TGG AAT ATG<br>GCT CTG GCT TCC TTC TCT TT
MARK2	IDT	Custom primer set	Fwd<br>Rev	AGA GAG TGC TAC GTG GTA AGT<br>GCT TGG ACG GAT TGA GGA TAA G
MARK3	IDT	Custom primer set	Fwd<br>Rev	GCT GGA TAT CAG TGA CCA GAA G<br>GAG CAG GTA AGT GGC TGT AAT
MARK4	IDT	Custom primer set	Fwd<br>Rev	AAT CGA AGT CAT GGT CGG TAT G<br>AGG AGC AGA TAG GTA GCA GTA A
SIK1	IDT	Custom primer set	Fwd<br>Rev	TCT CTG GAG GAA GAG CAA GA<br>CAA TGA TAC AGG GAG GGT TGA G
SIK2	IDT	Custom primer set	Fwd<br>Rev	CAG AAG CGT GAA GCC CAT AA<br>GCT GAC GGA AAG CTA CGA TAC
SIK3	IDT	Custom primer set	Fwd<br>Rev	TGA TGA TGT GCT ACT GGC TAT G<br>GGG AGT AGA TGG CGC TAT AAT G
PRKAA1	IDT	Custom primer set	Fwd<br>Rev	CCT CCT ACT CAA GCA CAA TGA T<br>CCT GGT GGT TTC GGT TAT AGA G

PRKAA2	IDT	Custom primer set	Fwd<br>Rev	CAT GGT TGT GCA TCG TGA TTT<br>CTC GCC GTC ACT CAT CAT ATT
IL11	IDT	Hs.PT.58.50431822	Fwd<br>Rev	CTC AGC ACG ACC AGG AC<br>GGA CAG GGA AGG GTT AAA GG
GAPDH	IDT	Hs.PT.39a.22214836	Fwd<br>Rev	TGT AGT TGA GGT CAA TGA AGG G<br>ACA TCG CTC AGA CAC CAT G

Supplementary Figure 1

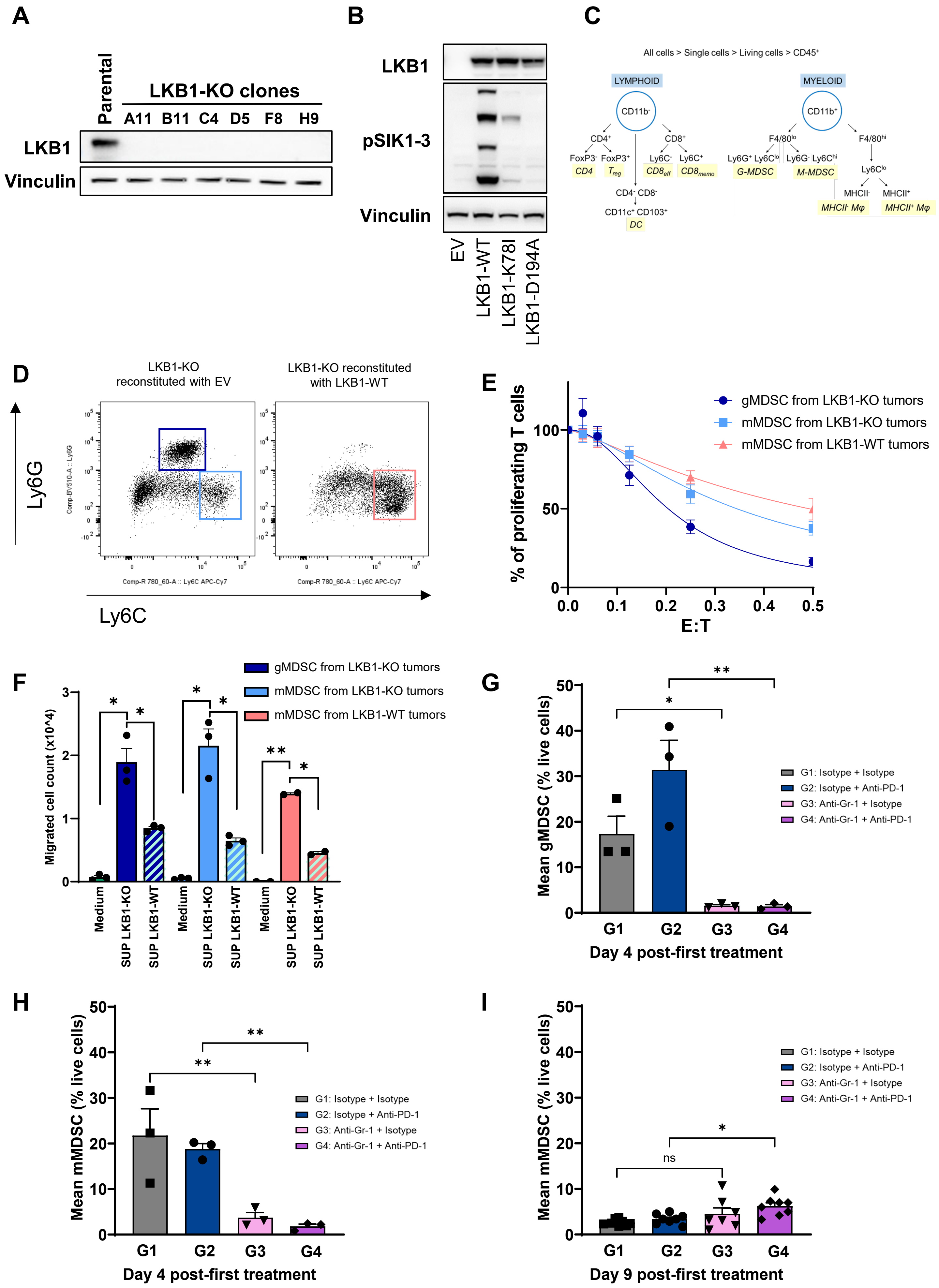

Supplementary Figure 2

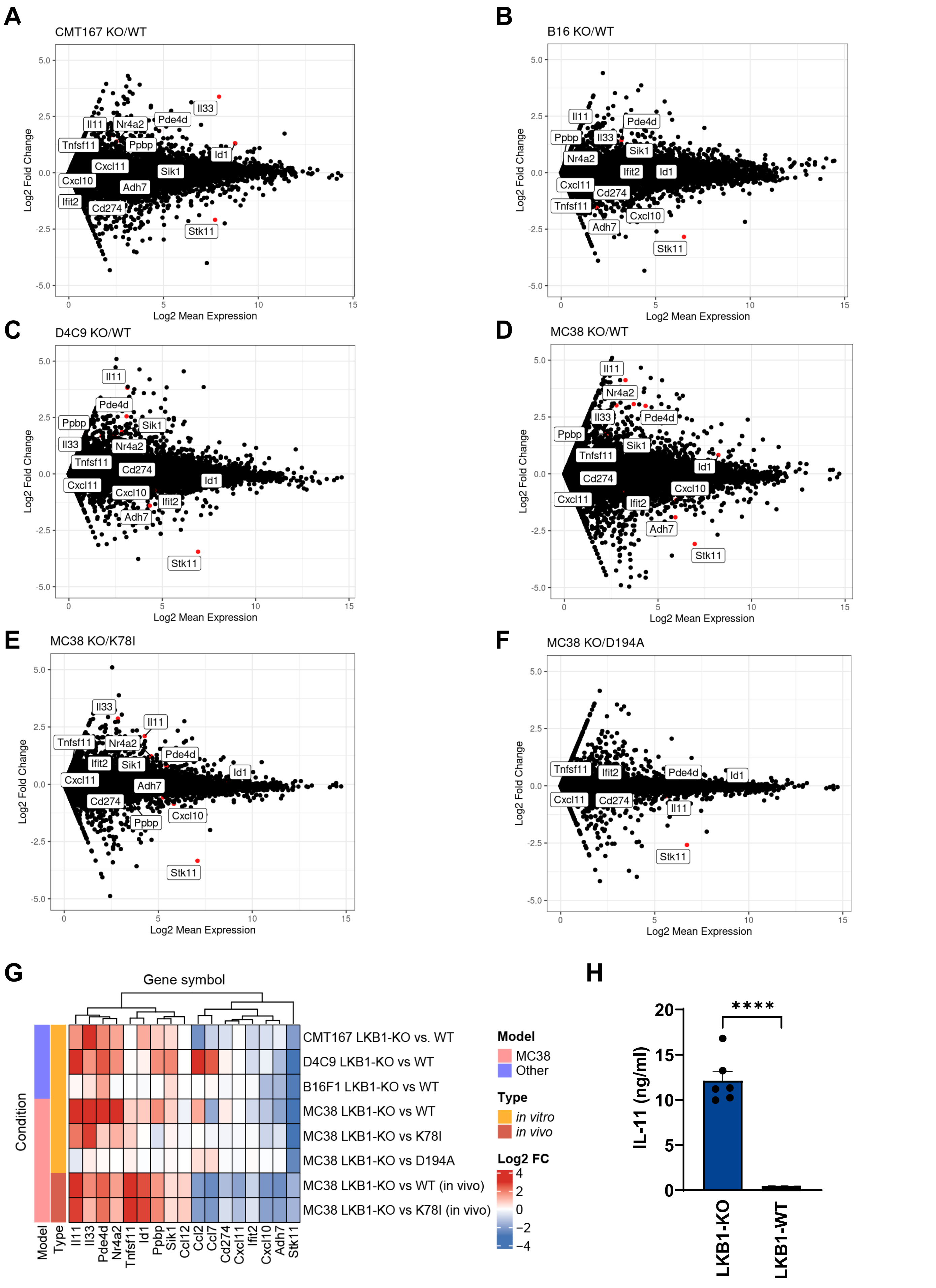

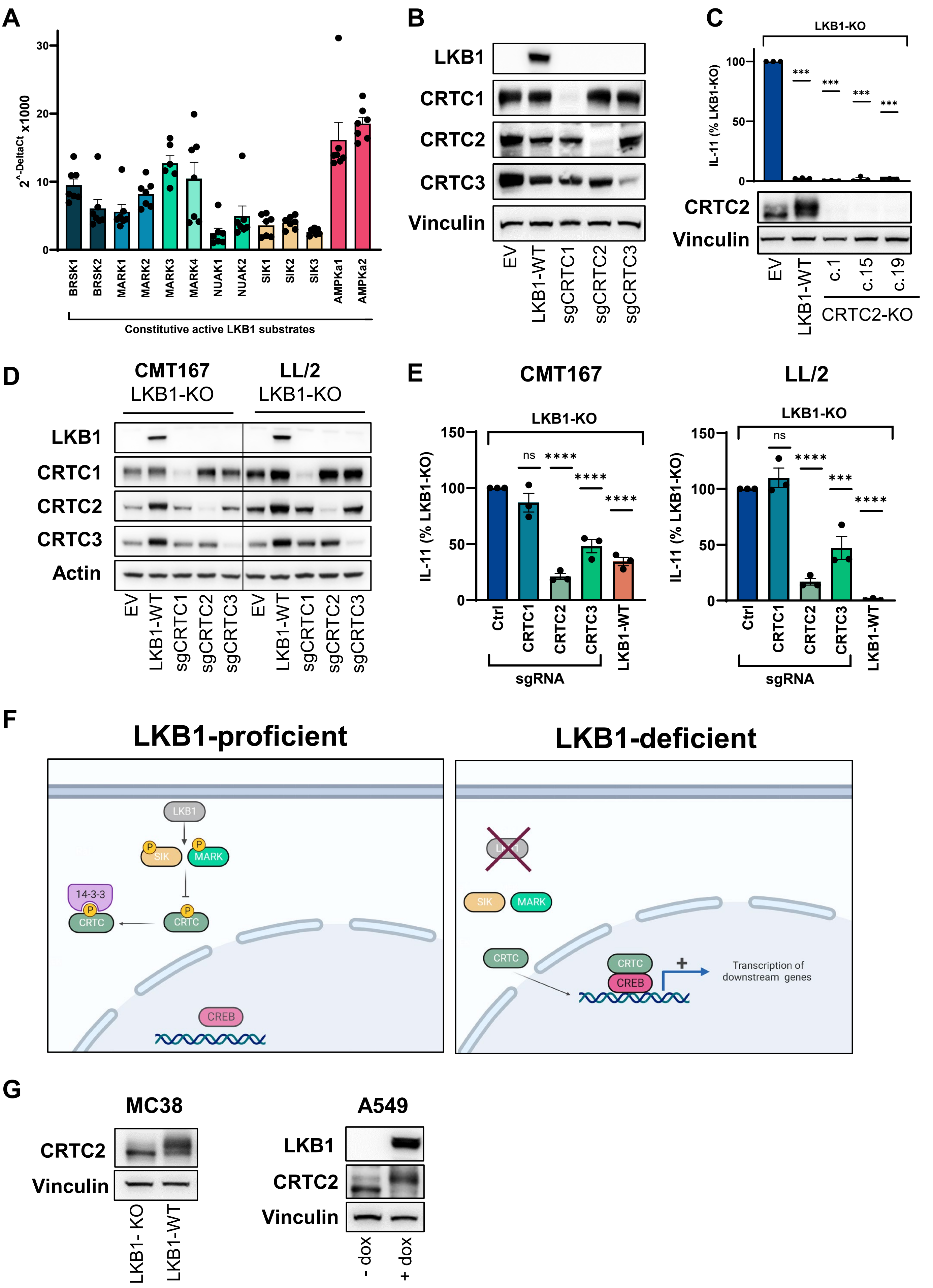
A
2^-DeltaCt x1000
BRSK1
BRSK2
MARK1
MARK2
MARK3
MARK4
NUAK1
NUAK2
SIK1
SIK2
SIK3
AMPKa1
AMPKa2
Constitutive active LKB1 substrates
B
LKB1
CRTC1
CRTC2
CRTC3
Vinculin
EV
LKB1-WT
sgCRTC1
sgCRTC2
sgCRTC3
C
LKB1-KO
IL-11 (% LKB1-KO)
CRTC2
Vinculin
EV
LKB1-WT
c.1
c.15
c.19
CRTC2-KO
D
CMT167
LKB1-KO
LL/2
LKB1-KO
LKB1
CRTC1
CRTC2
CRTC3
Actin
E
CMT167
LL/2
LKB1-KO
IL-11 (% LKB1-KO)
ns
Ctrl
CRTC1
CRTC2
CRTC3
LKB1-WT
sgRNA
F
LKB1-proficient
LKB1-deficient
LKB1
SIK
MARK
14-3-3
P
CRTC
CREB
Transcription of
downstream genes
G
MC38
CRTC2
Vinculin
LKB1- KO
LKB1-WT
A549
LKB1
CRTC2
Vinculin
- dox
+ dox

Supplementary Figure 4

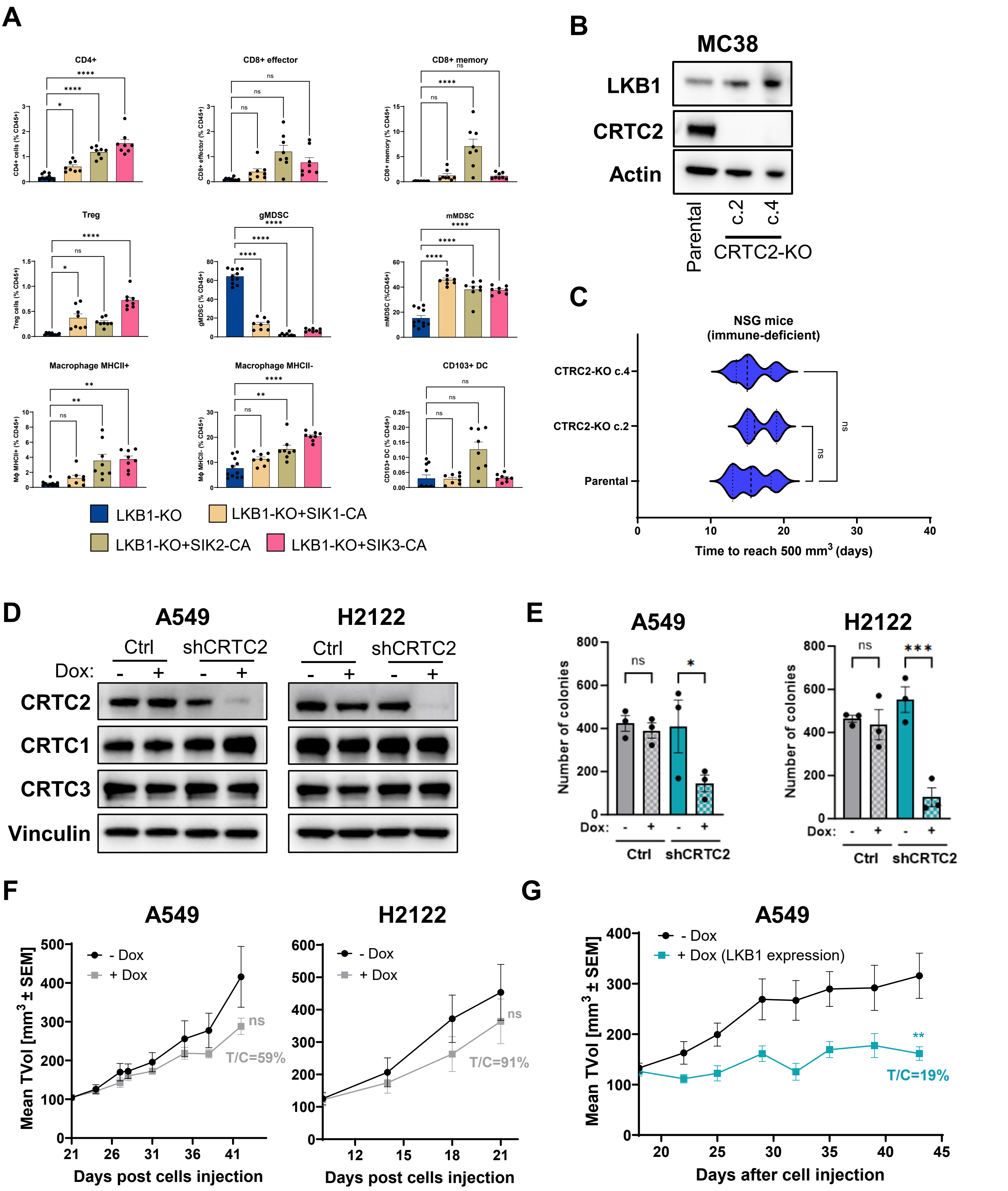

Supplementary Figure 5

A

Term	Overlap	P-value	Adjusted P-value	Odds Ratio	Combined Score
**Interferon Gamma Response**	19/200	1.16E-10	5.10E-9	7.54	172.42
**TNF-alpha Signaling via NF-kB**	16/200	4.10E-8	9.02E-7	6.17	105.02
**Xenobiotic Metabolism**	13/200	7.83E-6	1.15E-4	4.88	57.40
**Interferon Alpha Response**	9/97	1.21E-5	1.33E-4	7.12	80.60
**Fatty Acid Metabolism**	11/158	2.08E-5	1.83E-4	5.23	56.37

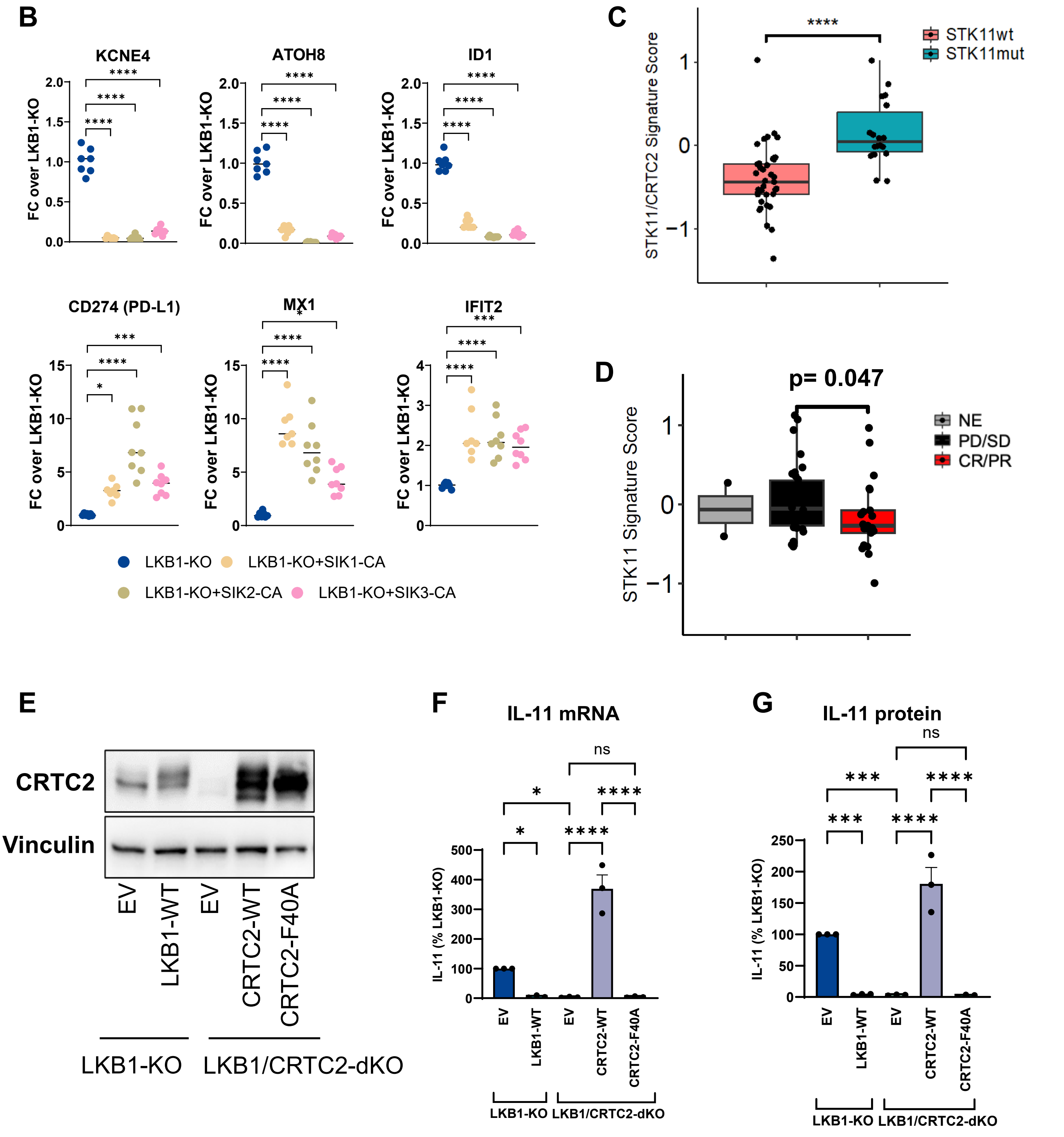

Supplement: Supplementary file 1 — Appendix 01 (PDF) [file pnas.2508762123.sapp.pdf]
